# Can real-world data really replace randomised clinical trials?

**DOI:** 10.1186/s12916-019-1481-8

**Published:** 2020-01-15

**Authors:** Sreeram V. Ramagopalan, Alex Simpson, Cormac Sammon

**Affiliations:** 1 London School of Economics and Political Science, Houghton St, London, WC2A 2AE UK; 2 Bristol-Myers Squibb, Sanderson Road, Uxbridge, UB8 1DH UK; 3 PHMR, Berkley Grove, London, NW1 8XY UK

**Keywords:** Real world data, Randomised clinical trials, Epidemiology, Treatment, Effectiveness

## Background

Classically, randomised controlled trials (RCTs) are considered the gold standard for demonstrating product efficacy for the regulatory approval of medicines. However, as personalised medicine becomes increasingly common, patient recruitment into RCTs is affected and – sometimes – it is not possible to include a control arm [1].

Real-world data (RWD) are data that are collected outside of RCTs [2]. They are gaining increasing attention for their use in regulatory decision-making. The United States twenty-first Century Cures Act mandated that the US Food and Drug Administration (FDA) should provide guidance about the circumstances under which manufacturers can use RWD to support the approval of a medicine. More recently, investigators from the European Medicines Agency (EMA) detailed their views on this topic [3].

### RWD for regulatory approval: opportunities and challenges

Eichler et al., from the EMA, state that, “the RCT will, in our view, remain the best available standard and be required in many circumstances, but will need to be complemented by other methodologies to address research questions where a traditional RCT may be unfeasible or unethical.” Thus, the gauntlet has been laid down for RWD to be used to support European regulatory approval. Indeed, RWD has been used by the EMA to approve several medicines for rare/orphan indications [4]. Eichler and colleagues, however, highlight that RWD methods must be critically appraised before they can be more widely accepted. They suggest that this appraisal can be undertaken via prospective validation of any proposed method with a pre-defined protocol.

Why the need for validation? Studies of the concordance between the results of RCTs and RWD studies investigating the same research question have given mixed results [5, 6]. It has been suggested that this discordance can be attributed to differences in the populations being investigated, or bias in RWD studies as a result of lack of randomisation.

Using an example of cancer risk in statin users, Dickerman and co-workers attempted to understand why RWD studies have shown a protective effect and RCTs showed no effect on neoplasm incidence [7]. One of the key principles of an RCT is to assess patient characteristics at baseline to check study eligibility based on inclusion/exclusion criteria. If eligibility is met, the next task is to randomise subjects into groups and, subsequently, to provide treatment as assigned for each group. Dickerman et al. operationalised a similar ‘target trial’ approach using RWD and followed up trial-eligible new and non-users of statins to compare rates of cancer between these groups. Performing the analysis in this way enabled the researchers to illustrate that results from RWD were in acquiescence with those from RCTs. Furthermore, previously reported differences were largely a result of two avoidable issues: immortal time and selection bias caused by the inclusion of prevalent statin users (prevalent users had to have survived without cancer up to baseline, leading to artificially lower rates of cancer in the statin group), rather than being attributed to the lack of randomisation per se.

As Dickerman et al. acknowledge, a limitation of the outcome they studied is that confounding by indication (whereby the reason for prescribing a patient medication is also associated with the outcome of interest) is unlikely to have a major role. Where the outcome is more likely to be affected by confounding by indication, then – to mimic the randomisation element of an RCT and appropriately compare treatment groups – RWD studies must carefully adjust for all baseline confounders. In this regard, Carrigan et al. recently report results exploring a research question more likely to be affected by confounding by indication [8]: whether control groups generated from RWD could approximate the control arms used in published RCTs in non-small cell lung cancer. In 10 of the 11 analyses conducted, hazard ratio estimates for overall survival derived from comparing RWD control arms with the intervention arm from the RCT were similar to those seen in the original RCT comparison. However, the analyses showed that a simple ‘target trial’ alignment of the RWD arm with the trial inclusion/exclusion criteria could not fully replicate the RCT effect estimate; additional adjustment to control for confounding using propensity scores was required. The single non-concordant analysis was thought to be associated with a biomarker that was likely enriched in the RCT but was not present in RWD and therefore could not be adjusted for. This exception to the overall consistency between RWD and RCT findings highlights the importance of needing RWD with information available on all possible confounders to avoid generating inaccurate results.

These two recent studies show that analytical methods and approaches are in place to enable consistency between RCT and RWD results. Further evidence will arise from the FDA-funded RCT DUPLICATE project, which will investigate RCT–RWD concordance on a larger scale [9]. In light of this, the question arises: how many examples are required before regulators can begin to accept RWD for regulatory decision-making? Eichler et al. state that the answer is unlikely to be simple: decision-makers should perhaps first accept RWD analyses for situations in which there is a relatively small impact (e.g. label expansion) and then gradually expand acceptability as confidence in the method grows.

## Conclusion

Accumulating evidence suggests that appropriately conducted RWD studies have the potential to support regulatory decisions in the absence of RCT data. Further work may be needed to better illustrate the settings in which RWD analyses can robustly and consistently match the results of RCTs and, more importantly, the settings in which they cannot match them. After careful consideration of the potential for bias, regulators can then determine when they would unequivocally accept RWD in place of an RCT. If studies based on RWD are ever to replace RCTs, regulators may need to accept that the cost of accelerating patient access to treatment carries a higher level of decision-making uncertainty than that with which they are familiar.

## Data Availability

Not applicable.
